# Zinc concentrations in teeth of female walruses reflect the onset of reproductive maturity

**DOI:** 10.1093/conphys/coaa029

**Published:** 2020-04-13

**Authors:** Casey T Clark, Lara Horstmann, Nicole Misarti

**Affiliations:** 1 Water and Environmental Research Center, University of Alaska Fairbanks, 1764 Tanana Loop, Fairbanks, AK 99775-5860, USA; 2 College of Fisheries and Ocean Sciences, University of Alaska Fairbanks, 2150 Koyukuk Drive, Fairbanks, AK 99775-7220, USA; 3 Joint Institute for the Study of Atmosphere and Ocean, University of Washington, 3737 Brooklyn Ave NE, Seattle, WA 98105

**Keywords:** Age at maturity, population biology, reproductive biology, trace elements

## Abstract

Age at maturity is an important parameter in many demographic models and, for some species, can be difficult to obtain using traditional methods. Incremental growth structures act as biological archives, recording information throughout an organism’s life and possibly allowing for the reconstruction of life history events. Concentrations of zinc (Zn) in animal tissues are known to be linked to life history, physiology and reproduction and may be retained in incremental growth structures. This study reconstructed lifetime Zn concentrations in teeth (*n* = 93) of female Pacific walruses (*Odobenus rosmarus divergens*) collected from 1932–2016. Zn displayed a characteristic pattern of accumulation, with a change point marking the beginning of a lifelong, linear increase in Zn concentrations. We hypothesized that this change point marks the onset of reproductive maturity. The age at which the change point occurred (age_cp_) was estimated by counting tooth cementum growth layers. These estimates closely matched literature values of timing of first ovulation in female walruses. Total number of ovulations (estimated from ovary corpora counts from paired tooth/ovary specimens) was closely related to reproductive lifespan (total lifespan – age_cp_; R^2^ = 0.70). Further, age_cp_ tracked changes in Pacific walrus population size as a proportion of carrying capacity, decreasing when the population was depleted by commercial hunting and peaking when carrying capacity was exceeded. This novel approach will aid walrus management, and is likely applicable to other species, offering a potentially powerful tool for research, management and conservation of wildlife populations.

## Background

Biological structures that grow incrementally act as archives of conditions experienced by an organism throughout its life (Outridge *et al.*, 1995). These are typically calcified tissues, such as coral skeletons, fish otoliths and mammalian teeth, but may also include woody structures, such as tree rings and keratinous tissues like scales, baleen and claws (Dodge and Vaišnys, 1980; Outridge *et al.*, 1995; Gemperline *et al.*, 2002; Vighi *et al.*, 2019). Trace element concentrations in these tissues can provide a range of information including environmental conditions (Alibert and McCulloch, 1997), animal population structure and movements (Secor *et al.*, 1995; Outridge and Stewart, 1999) and physiological information related to diet, growth and reproduction (Thompson *et al.*, 2003; Austin *et al.*, 2013). Seasonal or annual changes in these parameters can be investigated by measuring elemental concentrations across incremental growth structures, and examining multiple specimens can provide information about population-level changes across broader timescales (Alibert and McCulloch, 1997). Mammalian teeth are particularly valuable for reconstructing long term changes in trace element concentrations. Tooth cementum grows incrementally, with many mammals adding one wide, heavily calcified layer and one narrow, hypo-calcified layer each year [collectively referred to as a growth layer group (GLG); Laws, 1952; Fay, 1982]. This results in a banded structure similar to that of tree rings, which can be used to estimate the age of the animal (Laws, 1952). Trace elements are incorporated into the tooth cementum as it is grown, and, once there, remain unchanged for the remainder of the animal’s life (Outridge *et al.*, 1995). Thus, analysis of these growth layers can be used to reconstruct a lifetime record of trace element concentrations experienced by an animal.

The potential for trace element concentrations in mammalian teeth to provide information about life history traits, in particular reproductive parameters, could have major implications for research and management of wildlife populations. Traits such as age at maturity, age at first reproduction and reproductive interval are critical factors determining an animal’s fitness (Gadgil and Bossert, 1970; Stearns, 1976). At the population level, variation in these parameters plays a major role in determining abundance and population growth (Cole, 1954). For large, long-lived mammals, age at reproductive maturity and pregnancy rates may be among the most important factors regulating populations (Eberhardt, 1977). Furthermore, these life history traits may exhibit responses to both density-dependent factors, such as availability of food resources, predation and disease (Eberhardt, 1977; Abrams and Rowe, 1996) and to density-independent factors, including extreme weather events and habitat alteration (Gaillard *et al.*, 2000). Both the average age at maturity and average age at first reproduction tend to increase when competition for resources is high, for example, when a population approaches or exceeds carrying capacity (Boonstra, 1994). Conversely, these parameters typically decline in a population substantially depleted by predation or human hunting pressure (Proaktor *et al.*, 2007; Sergeant, 2011). These life history traits can thus provide information about population status. They are key components of most population models, and their accurate estimation is critical to wildlife conservation and management (Chivers, 1999; Gaillard *et al.*, 2003). For wildlife populations, estimates of these parameters are typically generated through direct observation and monitoring of known or marked individuals, through anatomical examination of hunted, harvested or captured animals or using other life history information, such as survivorship data. For species that live in remote habitats, exhibit cryptic life histories or are otherwise difficult to observe, however, application of these traditional methods is often unfeasible. The development of new tools for estimating these parameters is thus sorely needed to improve management of these species.

**Figure 1:**
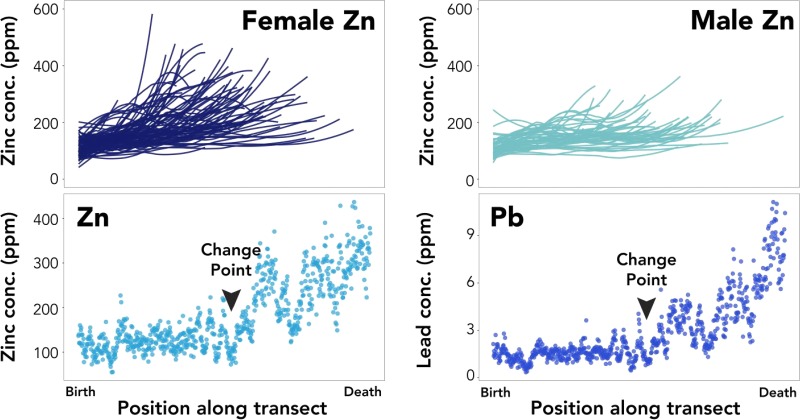
Top plots display lifetime trends in zinc concentrations (ppm) for female (left, dark blue) and male (right, light blue) walruses. Each line represents the loess-smoothed element concentrations across the lifetime of an individual walrus. X-axes indicate distance along the laser ablation transect (in mm), with zero (left side of graph) representing the inner edge of the cementum (birth) and the maximum values (right side of graph) representing the outer edge of the tooth (death). Bottom plots show typical patterns of zinc (left, light blue) and lead (right, dark blue) concentrations (ppm) for an individual female Pacific walrus along the laser ablation transect. Change points in element accumulation are noted on the plots.

**Figure 2:**
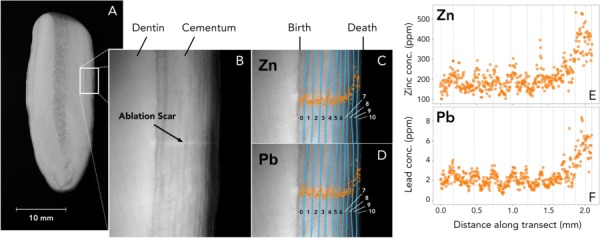
(**A**) Image of a cross-sectioned walrus tooth. (**B**) Image of the site of laser ablation, with annotations marking the dentin, cementum and ablation scar. (**C** and **D**) Image of the site of laser ablation, with the dark cementum layers highlighted in blue and the animal’s age in years denoted on each GLG. Zinc (**E**) and lead (**F**) concentrations (ppm) are overlaid on the images to show how the data align with the growth layers.

**Figure 3:**
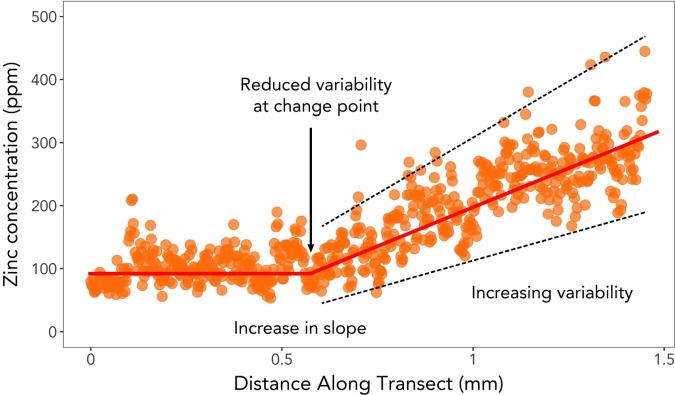
Characteristics of the change point in zinc and lead concentrations within the cementum of female walrus teeth. Change points were marked by low elemental concentrations and decreased variability, followed immediately by a change in slope and increased variability that typically continued for the duration of the animal’s life.

Rapid environmental change and an increase in the intensity of anthropogenic activities in the Arctic have led to concerns about the future status of the Pacific walrus (*Odobenus rosmarus divergens*) population (Garlich-Miller *et al.*, 2011). In addition to being key players in Arctic marine ecosystems, walruses are an important subsistence food resource for many Alaskan and Russian native communities, and the health of the walrus population is critical to maintaining food security. Walrus teeth are ideal candidates for trace element analysis, given their large size, broad cementum layer and abundance in museum collections. Thus, a pilot study was conducted to measure concentrations of a suite of trace elements in walrus teeth to determine whether any of these elements could be useful for reconstructing population structure, migratory movements or life history events. This research revealed distinct and divergent patterns of accumulation of zinc (Zn) and lead (Pb) in the cementum of male and female walruses (Fig. 1). Both sexes had similar elemental concentrations early in life; however, males typically exhibited little accumulation of either element until the later portion of their lives, when Zn and Pb increased slightly. In contrast, female walruses tended to have higher concentrations of both Zn and Pb throughout their lives, with accumulation increasing rapidly midway through life, creating a change point in the concentration data (Fig. 2). Change points were characterized by low concentrations of Zn and Pb, with little variability, followed immediately by an increase in both elemental concentrations and variability that typically continued until the animal’s death (Fig. 3).

Analysis of Zn concentrations in mammalian teeth may reveal important information about life history traits and reproductive physiology. Much of what is currently known about the role of Zn in the body comes from studies of rats, rabbits and primates, including some studies involving human subjects. Zn is critical for processes related to metabolism, growth and reproduction (Beigi and Maverakis, 2015). This essential trace element plays many important roles in the body, including protein synthesis, DNA and RNA replication and enzyme function (Walsh *et al.*, 1994; Beigi and Maverakis, 2015). The body’s Zn requirements are elevated during periods of rapid growth including infancy, sexual maturation and pregnancy (Salgueiro *et al.*, 2002). In females, Zn deficiency may disrupt the synthesis and secretion of follicle-stimulating hormone and luteinizing hormone, halt ovulation and cause abnormal ovarian development (Swenerton and Hurley, 1968, 1980; Shaw *et al.*, 1974). Thus, measuring changes in Zn concentrations across an animal’s life could possibly reveal information about the timing of life history events, including processes related to growth and reproduction. Few studies have examined Zn concentrations in the teeth of mammals, and even fewer have analyzed enough samples or collected data with sufficient temporal resolution to detect patterns of accumulation that might be reflective of these processes (e.g. Edmonds *et al*., 1997; Ando Mizobata *et al*., 2007).

In contrast to Zn, Pb is a non-essential element with widely demonstrated toxic effects. Pb and Zn exhibit many shared chemical properties, including competitive binding to metallothioneins, proteins responsible for much of the body’s uptake, transport and storage of metals (Nielson *et al.*, 1985). Because both elements bind to the same sites on these proteins (Nielson *et al.*, 1985), it is reasonable to assume that the similarities in their accumulation result primarily from changes in uptake (i.e. metallothionein concentrations), rather than dietary intake. The processes surrounding the uptake of trace elements from diet and their relationship to reproduction are not well understood. During periods of high demand, such as pregnancy, Zn uptake and storage appears to be elevated via increased production of metallothionein (Chan *et al.*, 1993), which would also be expected to increase Pb uptake (Nielson *et al.*, 1985). Calcium is another important element for ovarian function, and absorption from the gut increases during ovulation (Kitai *et al.*, 1985; Brommage *et al.*, 1993). High demand for calcium can increase metallothionein production, resulting in greater uptake of metals including Pb (Scheuhammer, 1996) and likely Zn. Concentrations of both elements were measured for this study; however, due to Zn’s physiological importance and its role in reproduction, we focused on this element when developing our hypotheses and interpreting the data.

Given the high Zn requirements associated with sexual maturation, as well as the importance of this element to the development of female reproductive organs, we hypothesized that the change point in Zn concentrations reflected the onset of reproductive maturity. In this study, we explore the validity of this hypothesis using three different approaches. First, estimated ages at which the change points occurred (age_cp_) in 93 female walruses were compared to published ages of first ovulation in this species. Second, for a subset of walruses (*n* = 16) for which both tooth and ovary specimens were available, the relationship between the total number of lifetime ovulations (estimated through corpora counts in ovaries) and reproductive lifespan (total age estimated from tooth GLGs – age_cp_) was investigated. Finally, because study samples included specimens collected from the 1930s to 2016 with relatively even temporal coverage, changes in the average age_cp_ over the last century were examined in relation to suspected changes in the size of the walrus population.

## Methods

### Trace element analysis and data processing

Teeth of 93 female Pacific walruses were on loan from collections at the University of Alaska Museum in Fairbanks, Alaska and the National Museum of Natural History, in Washington, DC. The year of collection for these specimens ranged from 1932 to 2016, with consistent sample coverage (≥10 individuals/decade) beginning in the 1960s and continuing through the present decade. Some specimens from earlier time periods were collected during scientific expeditions in the Bering and Chukchi seas throughout the Pacific walrus range, whereas the majority of more recent samples were taken as part of the Alaska Native subsistence harvests in the communities of Gambell and Savoonga on St. Lawrence Island, Alaska. Each tooth was cut longitudinally with a low speed, water-cooled saw using a diamond blade to create a 1.5 mm-thick cross section of the center of the tooth. This cross section was then affixed to a microscope slide and polished using a 3000 grit diamond smoothing disc on a rotary polishing wheel. Samples were rinsed thoroughly with ultra-pure water after polishing and allowed to air dry. The rinse/dry process was repeated immediately prior to analysis.

Trace element analyses were conducted in the Advanced Instrumentation Laboratory at the University of Alaska Fairbanks (UAF), Fairbanks, Alaska. Concentrations of ^66^Zn and ^208^Pb were measured using an Agilent 7500ce Inductively Coupled Plasma Mass Spectrometer (ICP-MS; fitted with a cs lens stack to improve sensitivity) coupled with a New Wave UP213 laser. Instrumental precision for the ICP-MS is reported at ±5%. ^43^Ca was used as an internal standard for these analyses, and all results are reported in parts per million (ppm). Measured element concentrations were compared with a United States Geological Survey microanalytical phosphate standard (MAPS-4), as well as a National Institute of Standards and Technology Standard Reference Material (NIST SRM 610). Accuracy and precision were estimated by comparing element concentrations measured during ablation of the reference materials (*n* = 348) with reported concentrations. Zn and Pb measurements were both accurate to within 1% of reported values, with a precision (±1 standard deviation) of ±4% and ±5%, respectively. Laser transects were ablated at a beam width of 25 μm, at 55% power, with a pulse frequency of 10 Hz and a transect speed of 5 μm/s. Dwell times were 0.02 seconds for ^43^Ca, 0.01 seconds for ^66^Zn and 0.15 seconds for ^208^Pb. Ablation was conducted at locations that maximized distance from the root, where cementum GLGs converge and become distorted, while avoiding areas of tooth wear near the crown, where not all cementum layers are present for sampling. Each transect was ablated from the cementum–dentin interface (first year of life) to the outer edge of the tooth (final year of life), thereby measuring lifetime changes in element concentrations for each animal.

Trace element data were extracted and processed in Igor Pro version 6.37 using the Iolite software package version 3.0. Statistical analyses were conducted using R version 3.4.1 (R Core Team, 2014) with RStudio version 1.0.153 (RStudio Team, 2015). Limits of detection were calculated for each analytical run using the standard method applied by Iolite (Longerich *et al.*, 1996). Typical limits of detection were 0.515 ppm for Zn and 0.047 ppm for Pb. Element concentrations falling below the limit of detection were replaced with a value of one half the limit of detection (US Environmental Protection Agency, 2000). Data points that were more than four standard deviations above or below the mean were considered outliers and removed from analysis (Tukey, 1977).

All specimens used in this study were obtained from museum collections and/or Alaska Native subsistence harvests, thus this work is Institutional Animal Care and Use Committee exempt. Specimens from subsistence harvests were transferred to UAF for analysis under a letter of authorization from the USFWS to Dr L. Horstmann.

### Tooth aging and change point assignment

After analysis on the ICP-MS, teeth were photographed under an optical microscope (Leica M165 C coupled with a Leica DFC295 camera) using reflected light. GLGs in the tooth cementum were counted and used to estimate the age of each animal (Fay, 1982; Garlich-Miller *et al.*, 1993). GLGs consist of paired cementum bands that typically accrete onto the tooth annually (Fay, 1982). Terminology surrounding GLGs has not been standardized and differences in the appearance of growth layers under reflected and transmitted light are a common cause for confusion. For the purposes of this study, the term ‘light layer’ refers to the opaque, hypercalcified layer that is built during periods of rapid growth and represents the period from approximately mid-April to mid-December (Supplementary Fig. S1). This layer appears white under reflected light but is dark under transmitted light. The term ‘dark layer’ refers to the translucent, hypocalcified layer that accretes during slow growth periods and represents the period from around mid-December to mid-April (Supplementary Fig. S1). This layer appears dark under reflected light, but allows light to pass through, so appears white when using transmitted light. Growth timing of dark and light cementum layers was based on estimates from the published literature (Fay, 1982) and confirmed by observations made on teeth used for this study. The manner in which animal ages are reported is another potential source for misunderstanding. Tooth ages are commonly reported as age classes, where an animal in the process of accreting its first GLG (light/dark 1) is considered to be age class 0, an animal growing the second GLG (light/dark 2) is age class 1 and so forth (Fay, 1982). All ages reported in this study follow this convention, with an age of ‘0’ referring to an animal in age class 0, ‘age 1’ referring to an animal in age class 1 and so on.

Each tooth was aged collaboratively by three observers, and the original GLG counts were revisited on at least two additional days to reassess previous age estimates. If the ages differed among any of the three counts, the median age estimate was used for analyses (Supplementary Table S1). Photographs were centered on laser ablation scars, and trace element data were overlaid directly on the image using Adobe Photoshop CS6. In this way, change points in Zn and Pb concentration data could be associated with an individual year in the life of a walrus. Change points were examined collaboratively by the same three observers who aged the teeth. The criteria for defining change points are outlined in the introduction and are visually displayed in Fig. 3. Change points were assigned based on the cooccurrence of the three criteria (decreased variability at change point, increased slope after change point, increased variability after change point) using both the Zn and Pb data. In instances where Zn and Pb indicated different change points, Zn was given priority. Each change point was assigned to an ‘A’, ‘B’ or ‘C’ category depending on the certainty with which it could be assigned to an individual year. ‘A’ indicated agreement among the observers and little to no uncertainty about the location of the change point. ‘B’ also indicated agreement among the observers but meant that a greater degree of uncertainty existed about the exact location of the change point (within 1 or 2 years). ‘C’ indicated that there was disagreement among the observers, a change point could not be detected or there were multiple possible change points that were more than 2 years apart. Change points assigned a ‘C’ were not used for subsequent analyses.

### Corpora counts and reproductive lifespan

To confirm the relationship between the change points and reproduction, we examined ovary pairs of 16 walruses for which tooth trace element concentrations had been determined by this study. Immediately following ovulation, the follicle that releases the ovum transforms into a structure called a corpus luteum (Supplementary Fig. S2). If the egg is not fertilized, the corpus luteum quickly degenerates, forming a small mass of scar tissue called a corpus albicans (Supplementary Fig. S2). If fertilization is successful, the corpus luteum is maintained, secreting progesterone throughout pregnancy. After pregnancy, the corpus luteum degenerates into a corpus albicans. In this way, evidence of each ovulation is preserved in the ovaries as corpora (Fay, 1982). Thus, the total number of corpora in ovary pairs provides an estimate of the number of ovulations over an animal’s life.

In this study, corpora in walrus ovaries were counted by an experienced observer. The number of corpora lutea and corpora albicantia for each walrus was summed to provide an estimate of the total lifetime ovulations for each animal. Linear regressions were used to compare these values to each individual’s total lifespan and reproductive lifespan. We defined reproductive lifespan as the number of years between an animal’s Zn (and Pb) change point and its death (total lifespan – age_cp_). The total number of corpora (i.e. ovulations) in a walrus’s life was expected to have a positive, linear relationship with the animal’s total age. Similarly, a walrus’s reproductive lifespan was expected to be positively correlated with total lifespan. Thus, for this analysis to provide support for the hypothesis that the change points are related to the sexual maturation, the regression using reproductive lifespan would have to substantially outperform the model using total lifespan to predict an animal’s number of corpora. The fit of the models was assessed by comparing R^2^ values. All data used for linear regressions were normally distributed.

### Changes in age_cp_ through time

Walrus teeth analyzed in this study are from animals that died between 1932 and 2016, thus presenting an opportunity to examine changes in age_cp_ during the last century. The year in which each animal was predicted to have attained reproductive maturity was estimated using tooth age and age_cp_. These estimates range from 1919–2006 (Supplementary Table S1). Walruses were then grouped into time periods based on suspected changes in the size of the walrus population documented in previous publications (Fay, 1982; Fay *et al.*, 1997; Garlich-Miller *et al.*, 2011; Speckman *et al.*, 2011; Taylor and Udevitz, 2015; Taylor *et al.*, 2018). Sample size considerations meant that earlier time periods were broader than more recent periods. This process resulted in nine separate time periods: 1910–29, 1940–59, 1960–69, 1970–79, 1980–89, 1990–94, 1995–99, 2000–04 and 2005–09. Differences in the average age_cp_ among these time periods were examined qualitatively and interpreted in relation to suspected historic changes in the size of the walrus population.

## Results

The estimated age at death (total lifespan) of walruses used in this study ranged from 2 to 28 years, with a median age of 14 years (Supplementary Table S1). Zn and Pb did not exhibit change points in three animals (ages 2, 3 and 5), likely because these animals had not yet reached reproductive maturity. Of the remaining 90 walruses, change points could be assigned with high (type A) or moderate (type B) confidence ~92% of the time. In ~8% of walruses analyzed, a change point could not be located despite the animal being old enough for one to be expected (12–19 years), or multiple possible change points were noted (type C). The average (mean and median) age_cp_ for walruses with identifiable change points was 5 years. The earliest change point occurred at 3 years and the latest occurred at 11 years.

**Figure 4:**
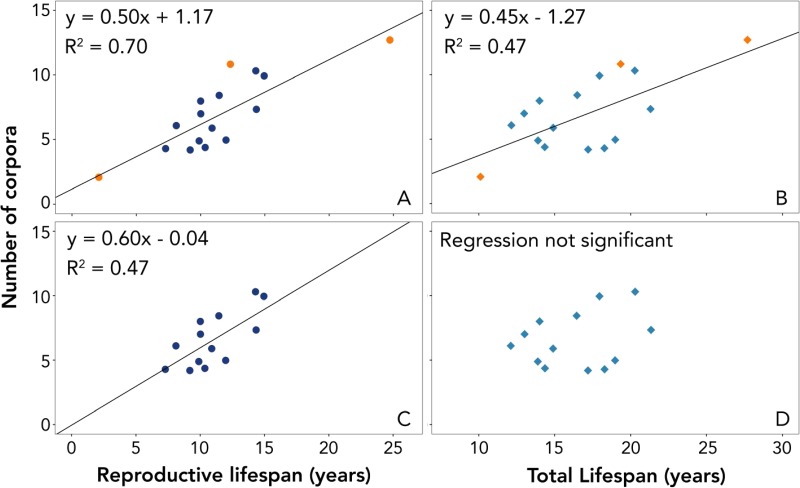
Regressions of number of corpora in walrus ovaries (total lifetime ovulations) against reproductive (**A**, **C**) and total lifespan (**B**, **D**). Top plots include all 16 available individuals with paired ovary and tooth data. Bottom plots show the same regressions with three individuals removed (two with change points with certainty code ‘C’ and one older animal with many corpora potentially acting as a leverage point). The greater R^2^ values of the regressions involving reproductive lifespan, as compared with those involving total lifespan, highlight the importance of age_cp_ in predicting the total number of ovulations a female walrus will have in her lifetime.

In this study, the total number of corpora in paired ovaries of individual walruses ranged from 2–13 (Supplementary Table S2). Most of these were corpora albicantia; however, the ovaries of seven individuals had corpora lutea, indicating these animals were pregnant (or had recently given birth) at the time of death. Regressing the number of total corpora against the reproductive lifespan (total lifespan – age_cp_) of each individual revealed a strong positive, linear relationship (F_1,14_ = 42.08, R^2^ = 0.70, *P* < 0.001; Fig. 4). This relationship explained more variability in the total number of corpora than did the regression of number of corpora against total lifespan (F_1,14_ = 12.41, R^2^ = 0.47, *P* < 0.01), indicating that age_cp_ is an important predictor of the total number of ovulations over the lifetime of a female walrus, and that the number of corpora is not simply a function of age. Two individuals in this regression had change points that were assigned with low certainty but were included in these regressions due to the scarcity of ovaries with associated tooth data. Additionally, one walrus was much older than the others (28 years), with many corpora in its ovaries, and was likely acting as a leverage point. To test the robustness of our results, the analyses were repeated with these three animals removed. This time, the number of corpora still had a significant, positive linear relationship with reproductive lifespan (F_1,11_ = 11.88, R^2^ = 0.52, *P* < 0.01), but the regression between number of corpora and total lifespan was not significant (F_1,11_ = 0.52, R^2^ = 0.04, *P* = 0.49).

**Figure 5:**
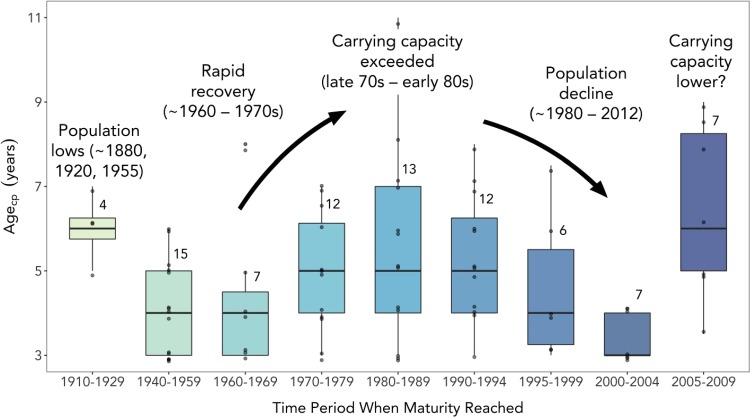
Annotated box plot of changes in age_cp_ of female Pacific walruses over the last ~100 years. Greater sample sizes in more recent years allowed for finer scale division of the data in later decades. Historical changes in the size of the walrus population are noted on the plot and are based on published literature (Fay, 1982; Fay *et al.*, 1997; Garlich-Miller *et al.*, 2011; Speckman *et al.*, 2011; Taylor and Udevitz, 2015; Taylor *et al.*, 2018). Note that average age_cp_ was lower during periods when the walrus population was depleted and higher when walruses were more abundant. Horizontal lines represent group medians, lower and upper bounds of boxes represent the 1st and 3rd quartiles of the data, respectively, and the whiskers represent the full range of the data. Data points more than 1.5 times the interquartile range above or below the median are considered outliers and are represented by individual data points. Sample sizes for each time period are denoted by numbers at the top right of each box.

Median age_cp_ ranged from ages three to six in discrete time periods during the last ~100 years (Fig. 5). Age_cp_ was very high in animals that reached maturity prior to the 1930s, though this time period was represented by only four animals, so this may be an artifact of sample size. The average age_cp_ was low for walruses reaching maturity from 1940–69, when the population was depleted and in the early stages of recovery. After 1970, average age_cp_ increased to a peak in the 1980s, corresponding to the period when the population exceeded carrying capacity and began to steeply decline. Age_cp_ then decreased to a low in the late 1990s and early 2000s. The average age_cp_ of walruses from the most recent time period (2005–09) was age six, one of the highest in the study period.

## Discussion

### Timing of first ovulation in walruses

The occurrence of first ovulation, or menarche, is an important stage in sexual maturation. In this study, age_cp_ closely corresponded with estimates from the published literature for the timing of first ovulation in Pacific walruses. The oldest reported age at first ovulation in Pacific walruses is 12 years (Krylov, 1966). Fay (1982) examined the timing of first ovulation in 205 female walruses and reported that the earliest ovulation occurred at the age of 4 years and the latest occurred at 10 years (Fay, 1982). Of these animals, none ovulated at age 3, ~11% had ovulated by age 4, ~44% by age 5, ~68% by age 6, ~89% by age 7 and ~96% by age 8. For walruses examined in the present study, no change points occurred by age 2, ~25% of animals exhibited a change point by age 3, ~49% by age 4, ~67% by age 5, ~80% by age 6, ~90% by age 7 and ~ 96% by age 8. This correspondence is compelling evidence that the change points in Zn and Pb are related to the onset of reproductive maturity (Fay, 1982). The small offset between these two studies may have resulted from differences between the timing of Fay’s sample collection and that of this study and is further discussed below.

Zn is critical to normal ovarian development, follicular growth and estrus (Favier, 1992; Bedwal and Bahuguna, 1994). The change points themselves are characterized by low elemental concentrations, with little variability and occur in the light layer of the tooth cementum (Fig. 3). This layer is grown between approximately mid-April and mid-December, a period that corresponds to blastocyst implantation, early stages of fetal growth and parturition (Supplementary Fig. S1). Ovulation and fertilization occur during the period when the dark cementum is developing (approximately mid-December to mid-April); therefore, any changes in Zn associated with these events would be recorded in the dark cementum layers. The location of the change point in the light layer of the cementum immediately preceding the increase in Zn concentrations thus suggests that the change point is likely representative of the period of reproductive development preceding an animal’s first ovulation, rather than the first ovulation itself.

The actual mechanisms driving the sustained, linear increase in Zn concentrations in the teeth of female walruses after the change point remain unclear. The importance of Zn in reproductive physiology is well established (Favier, 1992; Bedwal and Bahuguna, 1994); however, the numerous and complex roles this element plays in the body, ranging from cognition and olfaction to blood clotting and dark vision adaptation (Walsh *et al.*, 1994), make it difficult to isolate the most important ways in which Zn participates in reproductive physiology. The majority of the body’s Zn exists in metal-protein complexes, primarily metalloenzymes and metalloproteins, which use Zn as a structural element or an enzyme catalyst (Molokwu and Li, 2006). Zinc metallothionein is one such metalloprotein (Nielson *et al.*, 1985). Kidney metallothionein concentrations increase with age in rats (*Rattus norvegicus*), with females exhibiting higher metallothionein concentrations than males (Bremner *et al.*, 1981). Concentrations of both metallothionein and Zn increase with age in the liver and kidney of humans (Yoshida *et al.*, 1998) and harbor seals (*Phoca vitulina*; Tohyama *et al.*, 1986). Correlations between serum Zn and hormone concentrations have been reported for humans (Castro-Magana *et al.*, 1981; Favier, 1992; Prasad *et al.*, 1996; Hamza *et al.*, 2012). Zn plays an important role in skeletal development and is involved in the mineralization of bones and teeth (Ganss and Jheon, 2004; Stock *et al.*, 2017). The skeletal and reproductive systems are functionally linked, with bone morphogenic proteins playing a critical role in ovulation (Seifert-Klauss and Prior, 2010), thus it seems likely that the Zn change point observed in this study results from an interaction or, more likely, multiple interactions among the processes outlined above. The growth and reproductive development that occurs at the onset of sexual maturity likely results in increased demand for Zn and a subsequent increase in metallothionein production. It is possible that, as observed in humans and harbor seals, walrus metallothionein concentrations increase with age. Future work is needed to determine whether increased metallothionein and Zn concentrations affect bone and tooth mineralization and the amount of Zn incorporated into mineralized tissues.

Finally, it is possible that the patterns of accumulation observed in Zn and Pb result from a change in the growth rate or mineralization of the tooth cementum itself. In many mammals, cementum GLGs become narrower towards the edge of the tooth, corresponding with decreased body growth (Klevezal and Kleinenberg, 1969; Craighead, 1970; Bengtson and Siniff, 1981; Klevezal, 1996). Some species, including some marine mammals, exhibit a decrease in the width of the light cementum layers upon reaching sexual maturity or first reproduction (Bengtson and Siniff, 1981; Coy and Garshelis, 1992; Klevezal and Stewart, 1994; Klevezal, 1996), though this does not appear to be the case for harbor seals (Baker and Boveng, 1997). Slower cementum growth rates can result in changes in tooth mineralization, as evidenced by the dark and light growth layers produced each year. In this study, both Zn and Pb concentrations in walrus teeth tended to be higher in the dark layers and lower in the light growth layers, which can be seen as a cyclical signal in the calcium-normalized concentration data (e.g. Figs 1–3). This pattern likely results from differences in mineralization between the fast-growing, hypercalcified light layers and the slow-growing, hypocalcified dark layers (Stock *et al.*, 2017). Slower growth of the teeth later in life could thus be associated with changes in mineralization that lead to an increase Zn and Pb. In Pacific walruses, the thickness of the cementum layers typically begins to decline at around 2 to 3 years of age and may be associated with weaning rather than sexual maturity (Kryukova, 2017). Visual examination of the growth layer widths and the locations of the change points did not reveal an obvious correlation between the slowing of the cementum growth and age_cp_. Further, male walruses also exhibit declines in the width of cementum GLGs, but the animals examined in this study did not exhibit a change point in their Zn and Pb concentrations. Tooth growth rate and mineralization merit further consideration as factors contributing to the patterns of accumulation observed in Zn and Pb. Regardless of the mechanism responsible for generating the change points in Zn and Pb, the results presented here and in the other components of this study indicate that the timing of this phenomenon is related to maturation in female walruses.

### Corpora counts and reproductive lifespan

The ovary validation demonstrated that a walrus’s reproductive lifespan, estimated as the number of years between the Zn change point and the animal’s death, is closely related to the total number of ovulations occurring over the animal’s lifetime. This is the most direct evidence that the Zn change point is related to the onset of reproductive maturity and specifically to menarche. The slope and y-intercept of the linear relationship between reproductive lifespan and number of corpora provide further support for the validity of age_cp_ as a predictor of reproductive maturity. Given the long duration of walrus pregnancy (>1 year, Supplementary Fig. S1), the interval between ovulations is typically around 2 years (Fay, 1982). In the event of unsuccessful fertilization or failed pregnancy, animals might exhibit a one-year interval between ovulations, but such instances are likely balanced out by an increase in the ovulation interval later in life (Fay, 1982). The 0.5 slope of the regression line (Fig. 4) predicts that one new corpus is created for every 2 years of reproductive lifespan, thus matching the expected two-year ovulation interval. Because the reproductive lifespan is defined as beginning in the year where the change point (and thus, the assumed first ovulation) occurs, the y-intercept for the regression of reproductive lifespan and number of corpora should be 1. That is to say, a walrus in the first year of its reproductive lifespan should have ovulated once. The actual y-intercept from this regression was 1.17, again closely approximating the expected value. Many individual measurements were required to produce data for this regression (Zn and Pb elemental concentrations, tooth age estimates, change point estimates, ovary corpora counts), each incorporating some degree uncertainty and variation. Given these numerous sources of uncertainty, the variability in the total number of corpora explained by reproductive lifespan (R^2^ = 0.70) is remarkable and is perhaps the best support for our hypothesis that age_cp_ is an indicator of the onset of reproductive maturity.

Though modeled here using linear regressions, the relationships between lifespan (both total and reproductive) and number of corpora in the ovaries are expected to be non-linear. Variability in the age of first ovulation and an increasing ovulation interval in older animals would result in a sigmoidal curve for the relationship between total lifespan and number of corpora (Supplementary Fig. S3). Because the reproductive lifespan begins with first ovulation, the early portion of the sigmoidal curve is cut off, resulting in something resembling a negative exponential curve (Supplementary Fig. S3). In both cases, regression (fading) of corpora albicantia might contribute to the decreasing slope in older animals. The rate of regression is unknown for walruses, but in very old animals, corpora created early in life might become more difficult to see, thus may be less likely to be counted. Because the age at which senescence begins and the rate of corpora regression are unknown, it remains unclear at what age the slopes of the curves would be expected to begin decreasing; however, it appears that the animals examined for this study were still on the linear portion of the curve. Further investigations of the relationships between total lifespan, reproductive lifespan and number of corpora in the ovaries are warranted to better define these relationships.

### Changes in age_cp_ through time

A central tenet of population biology holds that the average age of sexual maturation in a wildlife population will vary in response to population density. When the density of animal populations is high, age at sexual maturity typically increases, fertility and juvenile survival decrease and the average age of reproductive females in the population increases (Boonstra, 1994; Sergeant, 2011). The converse is true for populations at low densities. Average age at maturity can thus be used as an indicator of population status (Eberhardt and Siniff, 2011), particularly with respect to the size of the population relative to available habitat and food resources. This concept may account for the small differences between the timing of first ovulation reported in Fay (1982) and the age_cp_ values in this study (Supplementary Fig. S4). When Fay collected data for his study, the walrus population was large and may have already exceeded carrying capacity (Fay *et al.*, 1989; Taylor and Udevitz, 2015; Taylor *et al.*, 2018). In contrast, the 93 walruses examined in the present study were collected between 1932 and 2016, thus our age_cp_ represents a longer-term average, encompassing periods when the population was at high and low densities. Given the expected relationship between population density and age at maturity, Fay’s walruses would therefore be expected to have reached menarche later than the animals sampled for this study.

Historic commercial hunting of Pacific walruses provides a unique opportunity to test the hypothesis that age at first ovulation varies in relation to the population density using the age_cp_ data. While incomplete and imprecise data make it difficult or impossible to accurately reconstruct historical walrus population trends (Speckman *et al.*, 2011), large-scale changes can be estimated (or at least conjectured, to quote Speckman and colleagues). Commercial walrus hunting is thought to have driven the walrus population to extremely low numbers in the 1880s, 1920s and 1950s (Fay *et al.*, 1989, 1997; Hufford and Loughlin, 2009). Hunting ceased in the 1950s, and the population recovered rapidly until the late-1970s, when carrying capacity was reached or exceeded (Fay *et al.*, 1997; Taylor and Udevitz, 2015; Taylor *et al.*, 2018). The population was in steep decline by 1981 and is believed to have continued to decline until ~2012 (Taylor and Udevitz, 2015; Taylor *et al.*, 2018). If the carrying capacity of the walrus population remained the same throughout this period, it is expected that the average age at reproductive maturity would track these changes in population size, decreasing when the population was depleted and increasing when walruses were abundant.

The median age_cp_ for walruses examined in this study closely tracked suspected changes in the status of the Pacific walrus population relative to carrying capacity over the last ~100 years (Fig. 5). The correspondence between the timing of the change point in Zn concentrations and documented lows and highs in walrus abundance is compelling and suggests that age_cp_ might be a valuable tool for monitoring population status. Because the average age at sexual maturity is expected to change in response to the availability of resources, stress levels, etc. (Boonstra, 1994; Proaktor *et al.*, 2007) and is not influenced only by the abundance of animals in a population, age_cp_ could be particularly useful as a management tool for walruses as arctic and sub-arctic marine environments change. Average age_cp_ increased substantially between the 2000–04 and 2005–09 time periods, jumping from the lowest value in the record to one of the two highest (Fig. 5). This may be an indication that the walrus population was again approaching carrying capacity at the end of the first decade of the 2000s, possibly driven by increased walrus abundance, environmental changes or other factors. The logistical challenges associated with estimating walrus abundance typically result in a great deal of uncertainty around abundance estimates and demographic parameters, making it difficult for managers to detect changes in size of the population (Chivers, 1999; Speckman *et al.*, 2011); however, the walrus population is believed to have been in decline until ~2012 (Taylor *et al.*, 2018). During this same period, climate change was altering the physical and biological features of walrus habitat in ways expected to reduce the availability and accessibility of walrus prey, thereby reducing the capacity of the environment to support large numbers of walruses (Garlich-Miller *et al.*, 2011). An elevated stress response associated with increased use of land-based haulouts and energetic costs associated with long transits to offshore foraging hotspots (Boonstra *et al.*, 1998; Garlich-Miller *et al.*, 2011; Jay *et al.*, 2011; MacCracken, 2012) may also have led to delayed maturity of walruses during the first decade of the 2000s. Determining the role of these and other factors impacting the status of the walrus population is challenging. However, the tendency for the average age at sexual maturity to reflect changes in the size of the population relative to carrying capacity makes the method presented herein a potentially valuable tool for assessing the status of the Pacific walrus population. Continued estimates of age_cp_ from animals harvested in recent years may provide critical information about the impacts of climate change and sea ice loss on the walrus population.

### Applicability to other species

It remains unclear whether the patterns of Zn and Pb accumulation examined in this study are unique to walruses. Zn concentrations in human and fin whale (*Balaenoptera physalus*) bone exhibit linear increases with age (Yoshinaga *et al.*, 1989; Vighi *et al.*, 2017). However, these results cannot be interpreted with respect to the Zn change point, as each individual is represented by only a single data point. Few studies have examined trace element concentrations across incremental growth structures at the resolution required to detect the Zn change point. Ando-Mizobata *et al.* (2006) measured the trace element concentrations in the tooth cementum of Steller sea lions (*Eumetopias jubatus*) Unfortunately, these authors were only able to examine element concentrations at a relatively small number of locations in the cementum (11–21, in contrast to hundreds or thousands in this study), thus were not able to achieve sufficiently high spatial resolution to capture any Zn change points in the teeth. Edmonds *et al.* (1997) measured Zn concentrations in the tusk of a female dugong (*Dugong dugon*; Edmonds *et al.*, 1997). Only one measurement was taken in each year of the animal’s life; however, the patterns of Zn accumulation appeared similar to those observed in walrus teeth in this study. That said, the apparent change point in dugong Zn data occurs at around age 25, substantially later than the expected age of ~10 years at sexual maturity for female dugongs (Marsh *et al.*, 1984). It thus seems reasonable to conclude that either the change in Zn accumulation is not associated with sexual maturation in dugong tusks or that the method used to age the tusk overestimated the age of the animal. Another study quantified Zn concentrations in desert tortoise (*Gopherus agassizii*) scutes, keratinous structures that grow incrementally over many years (Seltzer and Berry, 2005). Two of four tortoises studied (one male and one female) exhibited increases in Zn concentrations across the scutes (Seltzer and Berry, 2005) that were superficially similar to those we observed in walruses. The timescales represented by the laser transects in the tortoise study are, however, unclear, so it is difficult to say whether the patterns in Zn accumulation are associated with sexual maturity in tortoises. Finally, substantial and rapid Zn accumulation at sexual maturity in female squirrelfish (family *Holocentridae*) supports the idea that the pattern of increased Zn accumulation in association with sexual maturation might not be restricted to walruses or even to mammals (Thompson *et al.*, 2003).

## Conclusions

If the patterns observed in walruses exist in other species, the method for estimating age at reproductive maturity presented here has the potential to become a powerful tool for monitoring the status of wildlife populations. The ability to estimate age at reproductive maturity from mammalian teeth could greatly improve the management and conservation of wild populations. This potential is especially great for harvested or hunted populations, from which teeth are often recovered for aging purposes, and for free-ranging populations that are difficult to monitor in the wild. Additionally, this approach also makes it possible to conduct retrospective studies of changes in age at reproductive maturity using archived specimens, allowing researchers to gain a better understanding of past changes in wildlife populations. Examining archived historical, archaeological and paleontological specimens would allow for entirely new avenues of research. For example, this technique could potentially be used to estimate age at maturity in fossil specimens and extinct species or populations or to track changes in the average age at reproductive maturity in animal populations across thousands of years.

## Funding

This work was supported by the National Science Foundation Arctic SEES Program [grant number 1263848], with supplementary funds from the Bureau of Ocean Energy Management, Coastal Marine Institute, North Pacific Research Board, Cooperative Institute for Alaska Research and National Institutes of Health Biomedical Learning and Student Training Program [award numbers UL1GM118991, TL4GM118992 or RL5GM118990].

## Conflicts of Interest

The authors declare no conflicts of interest.

## Supplementary Material

clark_et_al_conservation_physiology_supplementary_information_coaa029
