# Supplementary material for: Zinc concentrations in teeth of female walruses reflect the onset of reproductive maturity
Source: Conserv Physiol. 2020 Apr 13;8(1):coaa029. doi: 10.1093/conphys/coaa029 (PMC7154182; doi:10.1093/conphys/coaa029)
Supplement: clark_et_al_conservation_physiology_supplementary_information_coaa029 [file clark_et_al_conservation_physiology_supplementary_information_coaa029.docx]

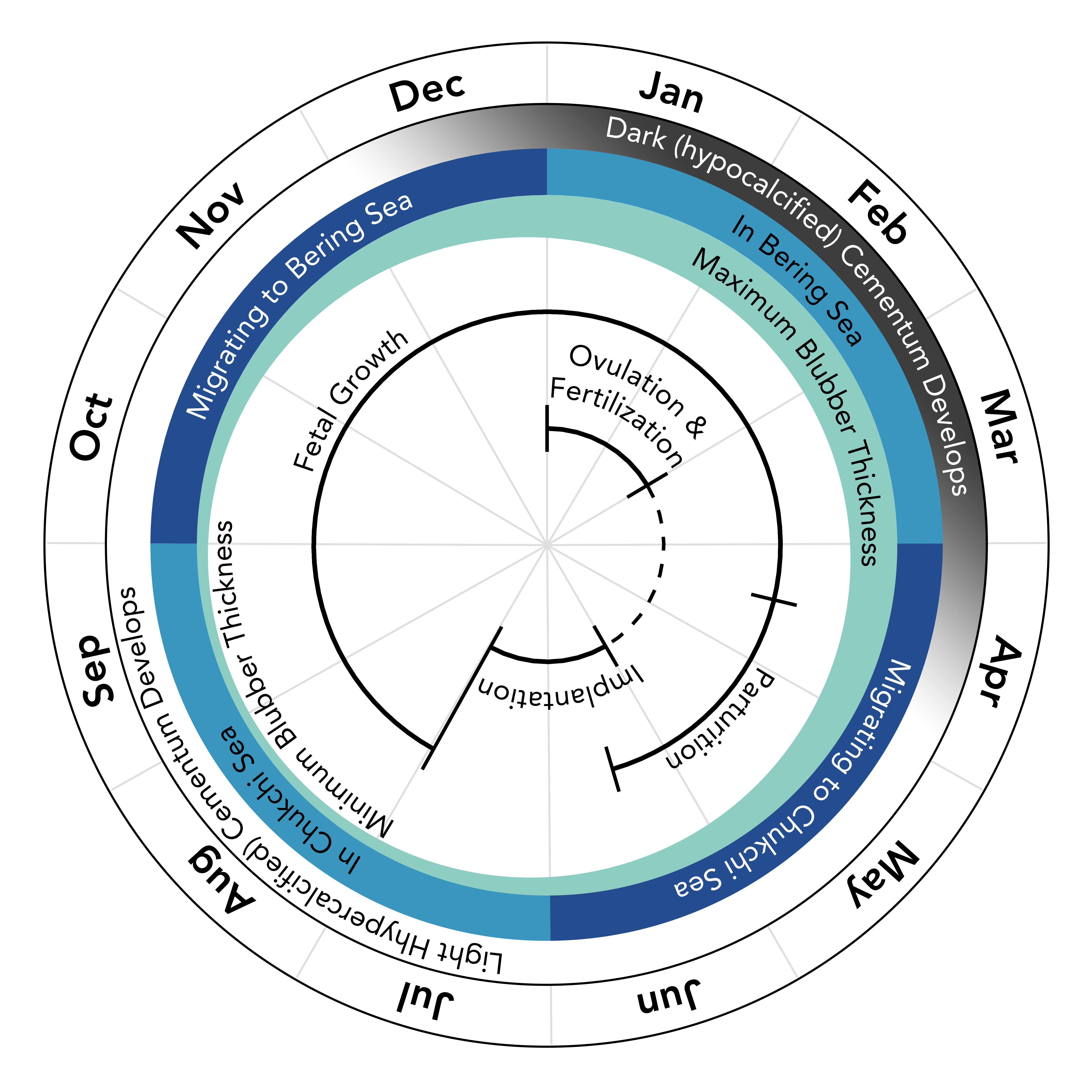


**Fig. S1.** Approximate timing of the annual cycle of female Pacific walruses. Outermost ring indicates month of year. Second ring (gray and white) represents expected timing of development of dark and light cementum layers. Third ring (dark and light blue) represents timing of annual migrations. Fourth ring represents relative blubber thickness. Innermost lines indicate timing of reproductive events (ovulation & fertilization, implantation, fetal growth, and parturition). Timings are based on estimates from the literature (Fay et al. 1997, Quakenbush et al. 1999) and, in the case of cementum growth, from observations made on the specimens used in this study.


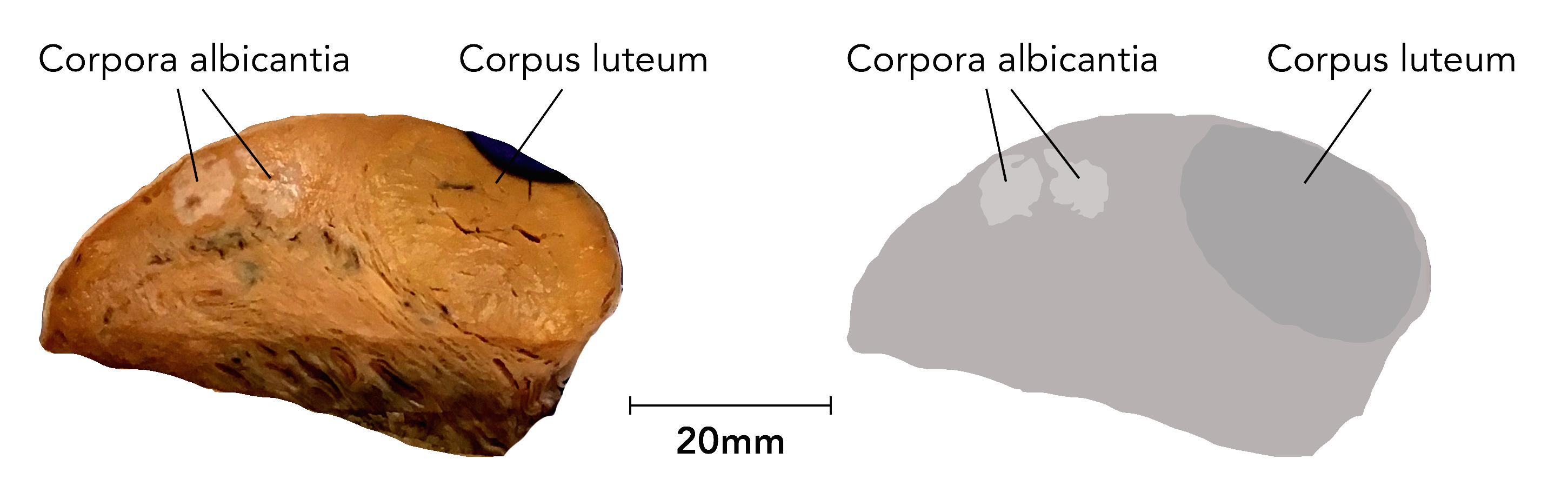


**Fig. S2.** Macroscopic cross-section of a walrus ovary, photographed (left) and as a diagram (right), with two corpora albicantia (small, light colored structures on top left of ovary) and one corpus luteum (large, yellowish structure on right side of ovary). Total corpora in walrus ovaries were counted to estimate number of lifetime ovulations for female walruses. Dark purple semicircle is the fingertip of the person holding the ovary.


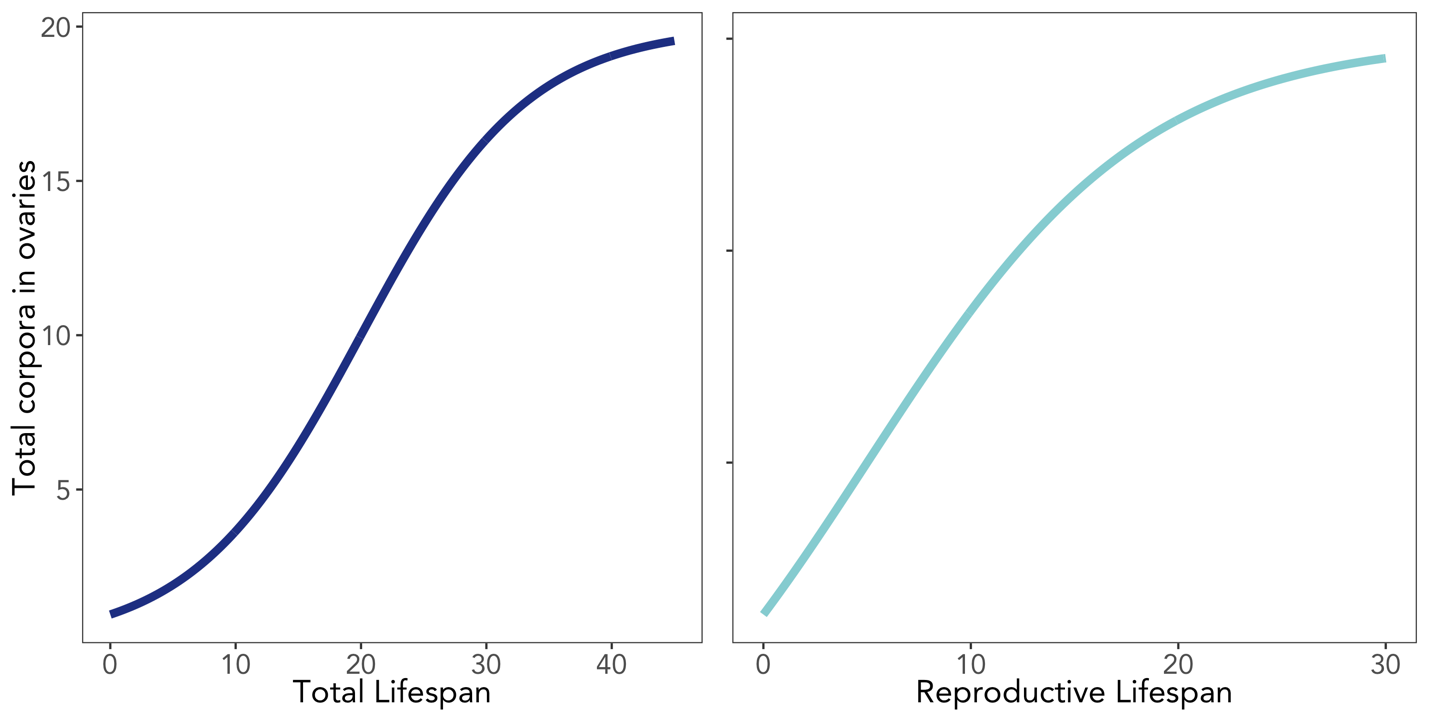


**Fig. S3.** Expected shape of the curves describing the relationships between the total number of corpora in the ovaries of a walrus (i.e., total lifetime ovulations) and total lifespan (left, dark blue) and reproductive lifespan (right, light blue). The sigmoidal shape of the curve in the left panel reflects the variability in the age of first ovulation (increasing slope in early life) and the increasing interval between ovulations (i.e., senescence) in older animals, as well as the possible regression of corpora albicantia in the ovaries (decreasing slope later in life). Reproductive lifespan begins with the first ovulation, thus does not exhibit the increasing slope in early life.


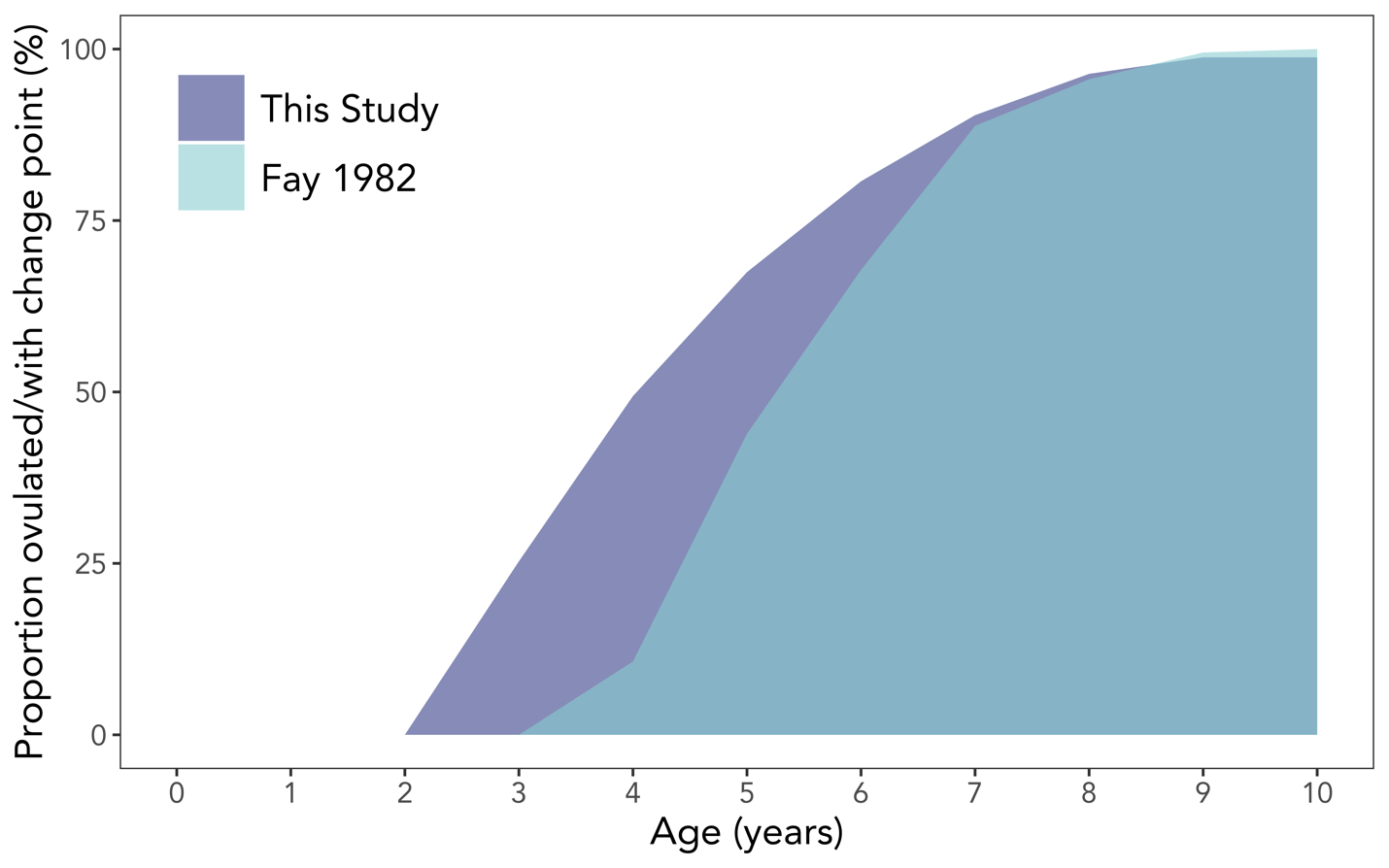


**Fig. S4.** Comparison of the timing of first ovulation, as reported by Fay (1982), and the appearance of change points in the tooth Zn and Pb concentrations of female walruses (this study). Proportion (%) of walruses that had ovulated (light blue, n = 205) or displayed a change point (dark blue, n = 93) is presented for age classes 0 – 10.

**Table S1.** Information for 93 female walruses examined in this study including project ID, University of Alaska Museum catalog number, median age (years), age_cp_ (years), certainty code for the change point (A, B, or C), collection year (year-at-death), cause of death, approximate birth year, and approximate year when change point occurred. Brackets around age_cp_ for specimens WAL936 and WAL948 indicate that these were low confidence (certainty code C) change points and were used only for the comparisons between number of corpora in ovaries and reproductive/total lifespan (due to low paired ovary-tooth sample size).

| Project  ID | Catalog Number | Median  Age | Age_cp_ | Change Point  Certainty  (A, B, C) | Year  Collected | Cause of Death | Approx.  Birth  Year | Approx.  Change Point  Year |
| --- | --- | --- | --- | --- | --- | --- | --- | --- |
| WAL023 | UAM:Mamm:16588 | 15 | 6 | A | 1933 | Research Collection | 1918 | 1924 |
| WAL025 | UAM:Mamm:11709 | 22 | 5 | A | 1970 | Subsistence Harvest | 1948 | 1953 |
| WAL033 | UAM:Mamm:10538 | 15 | 5 | A | 1973 | Unknown (Beachcast) | 1958 | 1963 |
| WAL036 | UAM:Mamm:5012 | 13 | 3 | A | 1954 | Unknown (Beachcast) | 1941 | 1944 |
| WAL045 | UAM:Mamm:16593 | 13 | 5 | A | 1932 | Research Collection | 1919 | 1924 |
| WAL048 | UAM:Mamm:11702 | 8 | 3 | A | 1956 | Subsistence Harvest | 1948 | 1951 |
| WAL055 | UAM:Mamm:11684 | 10 | 3 | A | 1972 | Subsistence Harvest | 1962 | 1965 |
| WAL056 | UAM:Mamm:11698 | 10 | 6 | A | 1959 | Unknown (Beachcast) | 1949 | 1955 |
| WAL059 | UAM:Mamm:11685 | 9 | 4 | A | 1972 | Subsistence Harvest | 1963 | 1967 |
| WAL062 | UAM:Mamm:11704 | 13 | 3 | A | 1958 | Subsistence Harvest | 1945 | 1948 |
| WAL063 | UAM:Mamm:11693 | 2 | No Change Point | - | 1966 | Subsistence Harvest | 1964 | - |
| WAL064 | UAM:Mamm:16591 | 18.5 | 6 | A | 1932 | Research Collection | 1913.5 | 1919.5 |
| WAL065 | UAM:Mamm:11691 | 10 | 4 | B | 1957 | Subsistence Harvest | 1947 | 1951 |
| WAL069 | UAM:Mamm:16586 | 19 | 7 | A | 1933 | Research Collection | 1914 | 1921 |
| WAL075 | UAM:Mamm:11686 | 7.5 | 3 | B | 1962 | Subsistence Harvest | 1954.5 | 1957.5 |
| WAL081 | UAM:Mamm:11689 | 3 | No Change Point | - | 1958 | Subsistence Harvest | 1955 | - |
| WAL121 | UAM:Mamm:16590 | 5 | No Change Point | - | 1933 | Research Collection | 1928 | - |
| WAL140 | UAM:Mamm:125315 | 22 | 7.5 | B | 2014 | Subsistence Harvest | 1992 | 1999.5 |
| WAL149 | UAM:Mamm:125328 | 16 | 8 | A | 2014 | Subsistence Harvest | 1998 | 2006 |
| WAL151 | UAM:Mamm:125287 | 15 | - | C | 2014 | Subsistence Harvest | 1999 | - |
| WAL153 | UAM:Mamm:125289 | 17 | 8.5 | B | 2014 | Subsistence Harvest | 1997 | 2005.5 |
| WAL155 | UAM:Mamm:125291 | 12 | 3.5 | B | 2014 | Subsistence Harvest | 2002 | 2005.5 |
| WAL282 | UAM:Mamm:129414 | 15 | 6 | A | 2015 | Subsistence Harvest | 2000 | 2006 |
| WAL630 | UAM:Mamm:99597 | 10 | 3 | A | 2005 | Subsistence Harvest | 1995 | 1998 |
| WAL780 | UAM:Mamm:131823 | 17 | 4 | A | 2016 | Subsistence Harvest | 1999 | 2003 |
| WAL781 | UAM:Mamm:131819 | 18 | 3 | A | 2016 | Subsistence Harvest | 1998 | 2001 |
| WAL783 | UAM:Mamm:131817 | 16 | 5 | A | 2016 | Subsistence Harvest | 2000 | 2005 |
| WAL784 | UAM:Mamm:181824 | 15 | 5 | A | 2016 | Subsistence Harvest | 2001 | 2006 |
| WAL787 | UAM:Mamm:131821 | 20 | 4 | A | 2016 | Subsistence Harvest | 1996 | 2000 |
| WAL809 | UAM:Mamm:130619 | 12 | 4 | A | 1987 | Research Collection | 1975 | 1979 |
| WAL810 | UAM:Mamm:130620 | 15.5 | 5 | B | 1987 | Research Collection | 1971.5 | 1976.5 |
| WAL811 | UAM:Mamm:130621 | 12 | 6 | A | 1987 | Research Collection | 1975 | 1981 |
| WAL812 | UAM:Mamm:130622 | 13.5 | 7 | A | 1987 | Research Collection | 1973.5 | 1980.5 |
| WAL813 | UAM:Mamm:130623 | 15.5 | 3 | A | 1987 | Research Collection | 1971.5 | 1974.5 |
| WAL814 | UAM:Mamm:130624 | 20 | 6.5 | B | 1987 | Research Collection | 1967 | 1973.5 |
| WAL828 | UAM:Mamm:108058 | 15 | 6 | B | 2006 | Subsistence Harvest | 1991 | 1997 |
| WAL843 | UAM:Mamm:128075 | 13.5 | 3 | A | 2012 | Subsistence Harvest | 1998.5 | 2001.5 |
| WAL844 | UAM:Mamm:128078 | 13.5 | - | C | 2012 | Subsistence Harvest | 1998.5 | - |
| WAL845 | UAM:Mamm:128086 | 12 | - | C | 2012 | Subsistence Harvest | 2000 | - |
| WAL846 | UAM:Mamm:130202 | 15 | 4 | B | 1981 | Subsistence Harvest | 1966 | 1970 |
| WAL848 | UAM:Mamm:108024 | 13.5 | 4 | A | 2006 | Subsistence Harvest | 1992.5 | 1996.5 |
| WAL852 | UAM:Mamm:90729 | 19 | - | C | 1979 | Subsistence Harvest | 1960 | - |
| WAL854 | UAM:Mamm:108129 | 15 | 5 | B | 1994 | Subsistence Harvest | 1979 | 1984 |
| WAL855 | UAM:Mamm:121084 | 17 | 5 | A | 1952 | Subsistence Harvest | 1935 | 1940 |
| WAL856 | UAM:Mamm:106394 | 13 | 3 | B | 1999 | Subsistence Harvest | 1986 | 1989 |
| WAL858 | UAM:Mamm:127992 | 11 | 9 | A | 2008 | Subsistence Harvest | 1997 | 2006 |
| WAL860 | UAM:Mamm:121109 | 14 | 4 | B | 1960 | Subsistence Harvest | 1946 | 1950 |
| WAL862 | UAM:Mamm:130694 | 13.5 | 5 | A | 1980 | Subsistence Harvest | 1966.5 | 1971.5 |
| WAL863 | UAM:Mamm:44164 | 12 | 4 | A | 1975 | Subsistence Harvest | 1963 | 1967 |
| WAL864 | UAM:Mamm:129607 | 14 | 4 | B | 1987 | Subsistence Harvest | 1973 | 1977 |
| WAL865 | UAM:Mamm:107472 | 12 | 5 | A | 2001 | Subsistence Harvest | 1989 | 1994 |
| WAL870 | UAM:Mamm:108259 | 22 | 7 | B | 1992 | Subsistence Harvest | 1970 | 1977 |
| WAL871 | UAM:Mamm:43591 | 14.5 | 8 | B | 1975 | Subsistence Harvest | 1960.5 | 1968.5 |
| WAL872 | UAM:Mamm:107986 | 18 | 7 | B | 2005 | Subsistence Harvest | 1987 | 1994 |
| WAL876 | UAM:Mamm:107053 | 16 | 5 | A | 1999 | Subsistence Harvest | 1983 | 1988 |
| WAL877 | UAM:Mamm:106958 | 14.5 | 6 | B | 2000 | Subsistence Harvest | 1985.5 | 1991.5 |
| WAL899 | UAM:Mamm:121047 | 14 | 4 | A | 1960 | Subsistence Harvest | 1946 | 1950 |
| WAL900 | UAM:Mamm:121060 | 8 | 4 | A | 1960 | Subsistence Harvest | 1952 | 1956 |
| WAL901 | UAM:Mamm:121145 | 14 | - | C | 1960 | Subsistence Harvest | 1946 | - |
| WAL902 | UAM:Mamm:121192 | 12 | 6 | A | 1960 | Subsistence Harvest | 1948 | 1954 |
| WAL903 | UAM:Mamm:121196 | 12 | 3 | B | 1960 | Subsistence Harvest | 1948 | 1951 |
| WAL904 | UAM:Mamm:121197 | 10 | 3 | B | 1960 | Subsistence Harvest | 1950 | 1953 |
| WAL905 | UAM:Mamm:130497 | 13 | 7 | A | 1981 | Subsistence Harvest | 1968 | 1975 |
| WAL907 | UAM:Mamm:43554 | 11 | 6 | A | 1975 | Subsistence Harvest | 1964 | 1970 |
| WAL908 | UAM:Mamm:43588 | 12.5 | 3 | A | 1975 | Subsistence Harvest | 1962.5 | 1965.5 |
| WAL909 | UAM:Mamm:43907 | 21 | 5 | B | 1975 | Subsistence Harvest | 1954 | 1959 |
| WAL910 | UAM:Mamm:44168 | 10 | 3 | A | 1975 | Subsistence Harvest | 1965 | 1968 |
| WAL913 | UAM:Mamm:129526 | 12 | 8 | B | 1987 | Subsistence Harvest | 1975 | 1983 |
| WAL915 | UAM:Mamm:129985 | 20 | 5 | B | 1987 | Subsistence Harvest | 1967 | 1972 |
| WAL917 | UAM:Mamm:106514 | 11 | 6 | B | 1999 | Subsistence Harvest | 1988 | 1994 |
| WAL918 | UAM:Mamm:106758 | 12 | 4 | B | 1996 | Subsistence Harvest | 1984 | 1988 |
| WAL919 | UAM:Mamm:107955 | 12 | 3 | A | 1993 | Subsistence Harvest | 1981 | 1984 |
| WAL920 | UAM:Mamm:108127 | 28 | 3 | B | 1995 | Subsistence Harvest | 1967 | 1970 |
| WAL922 | UAM:Mamm:108225 | 10 | 5 | B | 1996 | Subsistence Harvest | 1986 | 1991 |
| WAL923 | UAM:Mamm:108227 | 4.5 | 3 | B | 2000 | Subsistence Harvest | 1995.5 | 1998.5 |
| WAL924 | UAM:Mamm:107995 | 15 | 4 | A | 2007 | Subsistence Harvest | 1992 | 1996 |
| WAL925 | UAM:Mamm:107996 | 10 | 4 | A | 2007 | Subsistence Harvest | 1997 | 2001 |
| WAL926 | UAM:Mamm:127965 | 7 | 3 | A | 2008 | Subsistence Harvest | 2001 | 2004 |
| WAL927 | UAM:Mamm:127975 | 10 | 3 | A | 2008 | Subsistence Harvest | 1998 | 2001 |
| WAL934 | UAM:Mamm:107117 | 16 | 5 | A | 2002 | Subsistence Harvest | 1986 | 1991 |
| WAL935 | UAM:Mamm:108785 | 20 | 6 | B | 2000 | Subsistence Harvest | 1980 | 1986 |
| WAL936 | UAM:Mamm:108964 | 18.5 | (7) | C | 2002 | Subsistence Harvest | 1983.5 | - |
| WAL937 | UAM:Mamm:108984 | 21 | 7 | B | 2002 | Subsistence Harvest | 1981 | 1988 |
| WAL938 | UAM:Mamm:109020 | 15 | 4 | A | 2002 | Subsistence Harvest | 1987 | 1991 |
| WAL939 | UAM:Mamm:109028 | 15 | 4 | A | 2002 | Subsistence Harvest | 1982 | 1986 |
| WAL940 | UAM:Mamm:109059 | 17 | 3 | B | 2002 | Subsistence Harvest | 1985 | 1988 |
| WAL941 | UAM:Mamm:109077 | 13 | 4 | A | 2002 | Subsistence Harvest | 1989 | 1993 |
| WAL943 | UAM:Mamm:109152 | 18 | 11 | A | 2002 | Subsistence Harvest | 1984 | 1989 |
| WAL944 | UAM:Mamm:109212 | 14 | 4 | B | 2002 | Subsistence Harvest | 1988 | 1992 |
| WAL945 | UAM:Mamm:109221 | 17 | 8 | A | 2002 | Subsistence Harvest | 1985 | 1993 |
| WAL946 | UAM:Mamm:110003 | 19 | 7 | A | 2002 | Subsistence Harvest | 1983 | 1990 |
| WAL947 | UAM:Mamm:110177 | 13 | 3 | B | 2002 | Subsistence Harvest | 1989 | 1992 |
| WAL948 | UAM:Mamm:110238 | 10 | (8) | C | 2002 | Subsistence Harvest | 1993 | - |

**Table S2.** Information for walruses used in examinations of regressions of number of corpora and reproductive/total lifespan, including project ID, University of Alaska Museum catalog number, median age (years), total number of corpora, age_cp_ (years), certainty code for the change points (A, B, or C), and reproductive lifespan (years).

| Project  ID | Catalog Number | Median Age | # of Corpora | Age_cp_ | Change Point  Certainty  (A, B, C) | Reproductive  Lifespan |
| --- | --- | --- | --- | --- | --- | --- |
| WAL918 | UAM:Mamm:106758 | 12 | 6 | 4 | B | 8 |
| WAL920 | UAM:Mamm:108127 | 28 | 13 | 3 | B | 25 |
| WAL934 | UAM:Mamm:107117 | 16 | 8 | 5 | A | 11 |
| WAL935 | UAM:Mamm:108785 | 20 | 10 | 6 | B | 14 |
| WAL936 | UAM:Mamm:108964 | 18.5 | 10 | 7 | C | 11.5 |
| WAL937 | UAM:Mamm:108984 | 21 | 7 | 7 | B | 14 |
| WAL938 | UAM:Mamm:109020 | 15 | 5 | 4 | A | 11 |
| WAL939 | UAM:Mamm:109028 | 15 | 6 | 4 | A | 11 |
| WAL940 | UAM:Mamm:109059 | 17 | 9 | 3 | B | 14 |
| WAL941 | UAM:Mamm:109077 | 13 | 4 | 4 | A | 9 |
| WAL943 | UAM:Mamm:109152 | 18 | 4 | 11 | A | 7 |
| WAL944 | UAM:Mamm:109212 | 14 | 8 | 4 | B | 10 |
| WAL945 | UAM:Mamm:109221 | 17 | 4 | 8 | A | 9 |
| WAL946 | UAM:Mamm:110003 | 19 | 5 | 7 | A | 12 |
| WAL947 | UAM:Mamm:110177 | 13 | 7 | 3 | B | 10 |
| WAL948 | UAM:Mamm:110238 | 10 | 2 | 8 | C | 2 |
